# Initial diagnoses in university courses as an innovation in education—case study from Poland

**DOI:** 10.1016/j.heliyon.2022.e10633

**Published:** 2022-09-15

**Authors:** Elżbieta Jasińska, Michał Jasiński

**Affiliations:** a Department of Operations Research and Business Intelligence, Wroclaw University of Science and Technology, 50-370 Wroclaw, Poland; b Department of Electrical Engineering Fundamentals, Faculty of Electrical Engineering, Wroclaw University of Science and Technology, Wroclaw, Poland

**Keywords:** Diagnoses in education, Innovation in universities, Initial diagnoses at higher education, CSR to ecology and environmental protection issues

## Abstract

The need to identify the results to improve their quality is a mandatory practice in education. The scope and range of the indicated diagnoses vary at different stages of education. In elementary and secondary schools, the frequency of such practices is more intense compared to higher education. School diagnoses had to support processes to systematically communicate information to the environment about the status and level of education quality expressed with the results of the exams. Therefore, within school educational cycles, school diagnoses were incubated and developed, including initial diagnoses that create a supplement in the final stage (besides school grading). However, in higher education, such diagnoses need to be introduced and developed. Thus, this article proposes the methodology to perform initial diagnosis in higher education. In addition, a case study of the initial diagnosis performed at the selected Polish university was conducted. The presented example of diagnosis concerns the ecology problem and the awareness of the students under the corporate social responsibility approach. Additionally, diagnoses in mathematical problems are presented to present the continued diagnosis from the lower education stage.

## 1 Introduction–school diagnoses as the determinant and indicator of education quality in the school system

Diagnostics in education concerns itself with identifying the conditions, processes and effects of individual and group learning [1, 2]. It is based on models of learning, such as theoretical structures linking certain extrinsic and intrinsic variables of learning, on a variety of more or less sophisticated diagnostic methods, and on a mass of educational measurement tools [3]. The literature has generally pointed to two models of learning–the Vincent Okori model and the Benjamin Bloom model [4, 5]. The first is an instructional system model, involving the student, teacher, learning content, teaching equipment and school management. The second is the school learning model, based on cognitive preconceptual behavior, affective preconceptions, learning task(s), quality of instruction, level and type of achievement, affective effects and pace of learning [6, 7].

**Figure 1:**
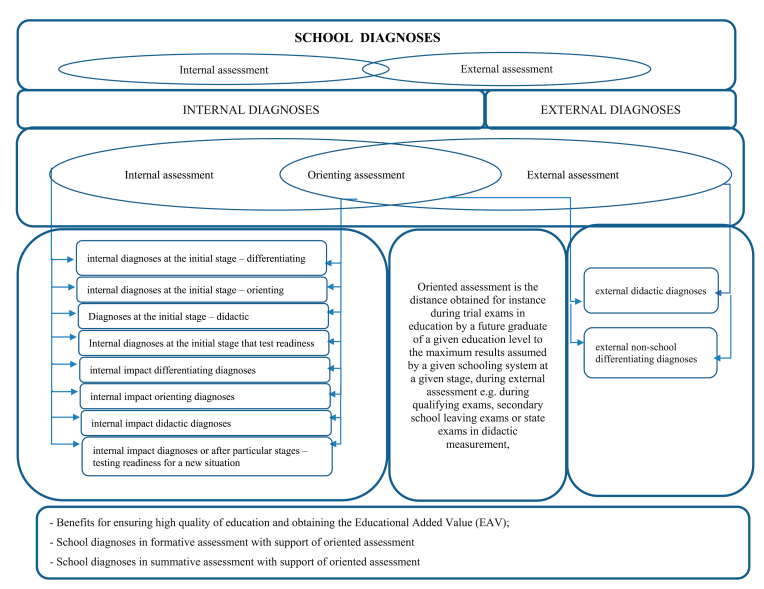
Incubation space for school diagnoses in the aspect (reality) of the process of school grading as: “a method supporting the assurance of high quality of education by applying oriented assessment that is developed on the basis of results of diagnoses”.

The need to diagnose learning results to improve their quality is a mandatory practice in education [8, 9]. The scope and range of these diagnoses vary at different stages of education [10]. At the lower levels of elementary and secondary schools, the frequency of such practices compared to higher education is more intense [11, 12]. This is related to a system of external assessment that covers state tests, middle school and high school graduation exams, as well as state exams that confirm professional qualifications at both the county and international level [13, 14]. The above-mentioned external assessment forced a particular reality in education for school diagnoses that on the one hand had to start serving its idea, provide help to future graduates in completing particular education levels or in studying their predispositions or competencies regarding a given subject towards further education (test after completing the sixth grade of an elementary school) or support the process of acquiring formal licenses for further education, i.e. at universities or schools or postsecondary schools (high-school leaving examination) [15, 16, 17]. On the other hand, the same school diagnoses also had to support processes of systematically communicating with the environment (i.e. to managers of an educational organization, parents of students) [18, 19], about the status and level of the quality of education [20] expressed with the results of education or expressed by the scope of developed competencies of students in the system, as expected by one, for their effectiveness. Hence, within school educational cycles, school diagnoses were incubated and developed, creating a supplement in the final stage (besides school grading) for high-quality results of education and often expressed with an education added value with varied effectiveness at the entry and when leaving of students in a given education system. Figure 1 presents the incubation space for school diagnoses in the space (reality) of the school classification process as: “a method supporting the assurance of the high quality of education by applying an oriented assessment developed based on the results of the diagnoses” [21, 22, 23].

As it can see the diagnosis is not a new area of research. It is known at the lower level of education [42, 43]. Hovewer in this article the need to extend the diagnosis to higher education level was indicated. Additionaly the case study of initial dignosis at University level was performed based on EU-country. The research object was located in Poland.

## 2 School diagnoses in the reality of higher education—status and shortages

The issue of the frequency, scope, and range of organized and conducted school diagnoses in higher education looks and develops differently compared to lower levels [24]. Recent research focuses on diagnoses at lower levels, for example:

• the information value of central school exams [25],
• development of a computer-based school exam application model for physic [26],
• factors influencing teachers' intentions and implementations [27] or motivation theories application [28] regarding formative assessment,
• analysis of secondary school internal scores versus national exams [29],
• the investigation of the questions mathematics teachers use on exams [30].

The phenomenon of diagnosis itself in the reality of universities as research centers is an elementary process in determining the state of defined scientific subjects in their selected research areas in resolving particular research problems. This is a different context and the usability of the diagnosis which in this sense originates from beyond the area of benefits in ensuring high quality of education, which are obtained by enforcing innovative practices consisting of school diagnoses in higher education.

Table 1 presents a comparison of experiments of the higher education environment with the experiments of the lower level education in the area of planning, organizing, and completing school diagnoses incubated in assessment systems, for benefits in ensuring high quality of education in those organizations.

**Table 1:** Comparing experiences in elementary and secondary education, and in higher education.

Types of assessments and diagnoses in the area of the elementary and secondary school system	Current state	Types of assessments and diagnoses in the area of the higher education	Current state
Formative assessment	Highly present	Formative assessment∗^11^	Highly present
Summative assessment	Highly present	Summative assessment∗^12^	Highly present
Oriented assessment∗^1^	Averagely present	Orienting assessment	Incidentally present
Internal assessment	Highly present	Internal assessment	Highly present
External assessment	Highly present	External assessment	None
School diagnoses	Highly present	School diagnoses (university)	Incidentally present
Internal diagnoses at the initial stage—differentiating∗^2^	Incidentally present	Internal diagnoses at the initial stage—differentiating.	None
Internal diagnoses at the initial stage—orienting∗^3^	Incidentally present	Internal diagnoses at the initial stage—orienting	Incidentally present
Diagnoses at the initial stage—didactic∗^4^	Highly present	Diagnoses at the initial stage—didactic	Incidentally present
Internal diagnoses at the initial stage that test readiness for learning a new associated subject∗^5^	Incidentally present	Internal diagnoses at the initial stage that test readiness for learning a new associated subject∗^13^	Incidentally present
Internal impact departmental differentiating diagnoses∗^6^	Incidentally present	Internal impact departmental differentiating diagnoses	None
Internal impact orienting diagnoses∗^7^	Incidentally present	Internal impact orienting diagnoses	Incidentally present
Internal impact diagnoses or after particular stages∗^8^	Incidentally present	Internal impact diagnoses or after particular stages∗^14^	Incidentally present
External didactic diagnoses∗^9^	Highly present	External didactic diagnoses	None
External non-school differentiating diagnoses∗^10^	Incidentally present	External non-school differentiating diagnoses	None

∗1. the distance obtained, for instance, during trial tests or exams in education by a future graduate of a given education level to the maximum results assumed by a given schooling system at a given stage, during external assessment e.g. during qualifying exams, secondary school leaving exams or state exams in didactic measurement. ∗2. Differentiating measurement is a measurement for competitions for talented students and those interested in a given discipline. ∗3. measurements for education results obtained later on in external diagnoses. ∗4 didactic measurements are completed to assess the level of knowledge, skills, and key competencies of students for the high quality of education, as well as the added value of education. ∗5. didactic measurement for students learning at a given education level, e.g. diagnosis of mathematics prior to learning physics. ∗6. differentiating measurements for particular stages, for example, contests. ∗7. didactic measurement for future results of education in external diagnoses. ∗8. testing readiness for a new situation, e.g. when starting secondary school after elementary school. ∗9. didactic measurements to define the quality of education in the school system and the possibility of improving this quality and determining the Education Added Value. ∗10. differentiating measurements for contest executed externally of a given educational organization. ∗11. during other forms of passing a subject, e.g. practical classes, laboratory. ∗12. when passing a lecture based on a formative assessment or an exam). ∗13. didactic measurements for students learning at a given education level, e.g. universities, diagnosis of statistics before learning “methodology of research with statistics”. ∗14. testing readiness for a new situation, when transferred onto a new level of higher education studies, e.g. 2nd or 3rd degree.

The proposed differentiation, the classification of diagnosis groups in the school (university reality) may be unclear to a lesser or larger extent, and may be the basis for more universal differentiations. This may depend on the needs and scale to ensure the quality of education as well as the possibility of its assurance. Furthermore, the terminology contained in the diagnosis may be a question of findings resulting from the character and specifics of a school facility where such diagnoses could be performed. On the other hand, practice or literature on this subject indicates that the place reserved for performing school diagnoses should be at lower levels of education, showing at the same time the shortages in this regard or a description of incidental practices of diagnoses, specifical shortages of initial diagnoses. Especially since the process of diagnosing the state of quality at the initial stage of education makes it possible to improve it and achieve the top level while retaining this state thanks to diagnoses performed along the way until the last stage of education. Then, this quality of education would be improved in the next education cycle. The same literature also presents a lack of appropriate diagnostic tools for this type of impact of academic teachers on their professional practice. Therefore, there is an urgent need to create organizational and executional solutions for school diagnoses at the higher education level due to their impact and to ensure high quality of education. For instance, presenting the state of knowledge of students at the initial stage thanks to school (university) diagnoses as well as their readiness for completing particular courses within their studies at universities, enables academic lecturers to choose proper teaching methods, applying innovative solutions for improving the diagnosed shortages, etc. It allows applying such professional, methodological, organizational, execution, and innovative tools in work with students that will result in ensuring high quality of education at universities and perhaps finding scientific talents among students [31].

## 3 Diagnoses in didactic measurement recognizing knowledge, skills, key competencies, and readiness of students—practices from selected polish university to ensure the quality of education

The literature on this subject lists diagnoses within the scope of didactic measurement [8] that determines the results of education, and after diagnosis, it is used to prepare and implement remedial and improvement programs and maintain quality. It also lists the differentiating measurement (that could also be a didactic measurement) but based on data originating from results of educating talented students that are found in various subject-related contests and competitions regarding various disciplines. Regardless of whether the diagnosis relates to the quality of education specified for the student population completing the basic school curriculums (didactic measurement) or for the student population executing contest programs that are beyond standard education processes, the quality aims at improving the existing state regarding benefits of ensuring high quality of education in schools and universities. When it comes to the latter ones, namely universities, the frameworks of the above-mentioned quality may be described differently in university practice, e.g. in subject sheets that can ensure such quality using the content included on them regarding the fact of completing a subject. Such sheets contain elements in their structure (i.e. initial requirements regarding knowledge, skills and other competencies, aims of the subject; student workload; subject effects of education; curriculum content; grading criteria; achievements of the subject education effect; the format of classes; applied didactic methods, didactic tools, etc.) that can be a starting point for the range and scope of diagnosis at the initial stage, e.g., to recognize students' readiness in didactic measurement, specifying the scope of knowledge, skills and their starting competencies relating to a given subject. Within the scope of the listed postulates, for school diagnoses, there were efforts to complete such diagnoses at universities.

### 3.1 Methodology of performing diagnoses in higher education

**Figure 2:**
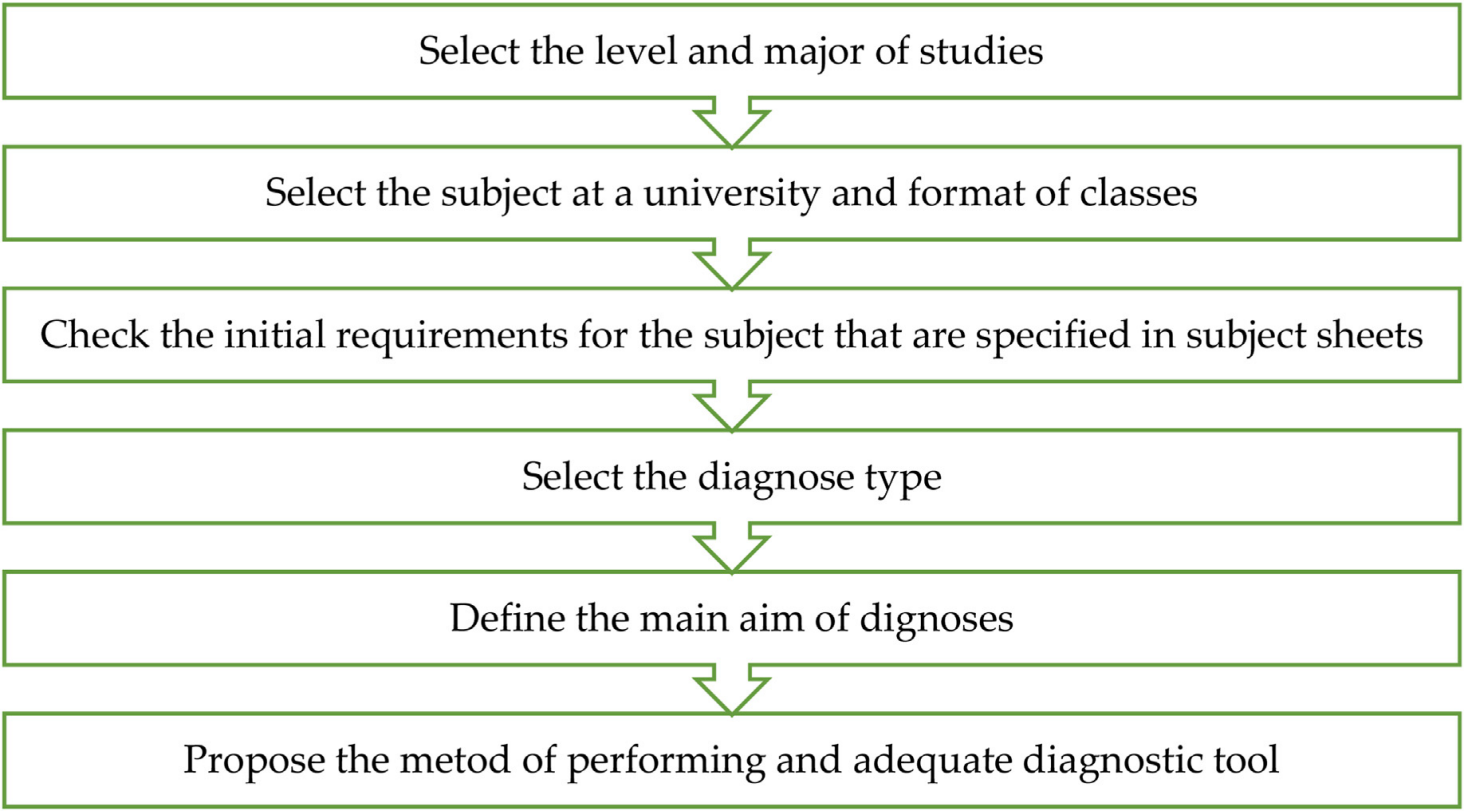
Methodology to perform the diagnoses in higher education.

The proposed step-by-step methodology to perform diagnostics in higher education is presented in Figure 2. It is based on the selection of the subject (course) for the level and major of the studies. Then the next step is to check the initial requirements, which are indicated in subject sheets, that are obligatory and deeply check by university councils. After this, an adequate diagnosis type is selected to obtain its goal. At this stage, different diagnoses may be selected, e.g., diagnoses at the initial stage—didactic, internal diagnoses at the initial stage—orienting, internal impact didactic diagnoses, internal impact orienting diagnoses, etc. Then based on the type of diagnosis and its aim, an adequate method of performing and recognizing tool must be selected.

### 3.2 Case studies from Poland

Based on the proposed methodology, the case study of the possibilities of performing diagnoses in a university was carried out. The university investigated is located in Lower Silesia, Poland. The performing of diagnoses was realized in accordance to University restrictions and recommendation as well as statutory goals. Students who participate in such diagnosis, attend them voluntarily and with anonymization of the results. Due to such assumptions no additional ethics agreement were settled. The selection of the subject of interest was based on the teaching experience of the article authors, which was obtained in the range of 2011–2017. The investigated subjects are as follows:

• Mathematics (lecture and practical classes)
• Mathematics with statistics (lecture and practical classes)
• Methodology of research with statistics (practical classes)
• IT tools in management (laboratory)
• Environmental protection (lecture)
• Ecology (lecture, project)
• Introduction to technical science (lecture)

The results obtained for the Polish University investigated for the indicated courses are presented in Table 2.

**Table 2:** Selected examples of the possibilities for completing university diagnoses in 2011–2017 at a university in Poland.

Level and major of studies	Subject*	Initial requirements**	Diagnoses type	Aims diagnoses, method and tools
Engineering studies, bachelor studies e.g.:	- Mathematics (lecture and practical classes) - Mathematics with statistics (lecture and practical classes)	Content regarding mathematics and statistics in the curriculum of a secondary school	Diagnoses at the initial stage—didactic	Measurement completed assessing the level of knowledge, skills, and key competencies of students for the high quality of education after completed studies.
-Management in Public Policies.			Internal diagnoses at the initial stage—orienting	Measurement recognizing readiness for learning mathematics and statistics at a higher level, e.g. during higher education
- Management.			Internal impact didactic diagnoses	Orienting measurement of students (constituting a forecast for them) regarding passing a mathematics and statistics exam at the end of a course
- Logistics.			Internal impact orienting diagnoses	Measurement enabling diagnosis of students after every semester, if the subject was planned during a bigger number of semesters and/or after each study level
- Mining and geology			Diagnoses completed during the first classes. The diagnostic tool consists of sets of open mathematics exercises at the level of secondary school or as a continuation of an exercise from previous semesters and levels	
Master's studies e.g.: - Finance management	- Methodology of research with statistics (practical classes)	No preliminary requirements were defined	Internal diagnoses at the initial stage that test readiness for learning a new associated subject,	Measurement completed for determining the level of knowledge regarding statistics, combinatorics, probability before learning “methodology of research with statistics” as a necessary component for a student completing a given subject.
- Administration management			Diagnoses completed during the first classes. The diagnostic tools are composed of sets of exercises regarding statistics, combinatorics, and probability.	
Engineering studies, bachelor studies e.g.: -Management in public policies	- IT tools in management (laboratory)	Content regarding IT tools in the curriculum of a secondary school	Internal diagnosis at the initial stage that test readiness for learning a new associated subject	The measurement was completed to define the scope of awareness of future managers in the impact made by them, from the point of view of caring about social benefits, based on their life experiences acquired thanks to being present at a global mining company as well as awareness of usability of innovative tools in management for business and social results.
- Management				
- Logistics			Diagnoses completed during the first classes. The diagnostic tool consists of global, European, and Polish models of CSR standards.	
Bachelor studies e.g.: - Management in Public Policies	-Environmental protection (lecture)	- biology - chemistry - technical science	Internal diagnoses at the initial stage that test readiness for learning a new associated subject.	The measurement was completed to define the sensitiveness of people studying at a given education level in the scope of environmental protection or ecology, regarding the needs of the natural environment in their future professional activity
	- Ecology (lecture, project)		Diagnoses completed during the first classes. The diagnostic tool consists of global, European, and Polish models of CSR standards; areas regarding environmental protection.	

* Name of a subject at a university and format of classes ** Specified in subject sheets.

### 3.3 Diagnoses for ecology and environmental protection

To discuss in more depth, an example of initial diagnoses in point of the environment is presented. Initial environmental diagnoses were made for ecology subjects in the first year of engineering studies, as well as environmental protection for students in the third year. These two populations were tested with the same diagnostic tool. The tool was obtained from a system of corporate social responsibility (CSR) models [32] that includes numerous criteria for ecology and environmental protection standards in managerial impacts [33, 34]. The model was based on [35, 36] The task of the students during the initial analysis was to analyze the model system provided and to highlight the criteria in them that could be the basis of the content for ecology and environmental protection. Students, using the knowledge obtained at lower education levels on the listed subjects in a varied way and at different levels, marked these criteria applying a different level and readiness status to complete a course on ecology and environmental protection in the listed studies. The marked and selected criteria in diagnosis at the initial stage created a list of standards only for ecology and environmental protection because, under the CSR doctrine, they are only a certain part of a broader range of the areas of its interest [37, 38]. The students studied, after the diagnosis was completed, presented lists of the selected criteria with various scales and ranges, because their knowledge of ecology varied. For instance, in the case where a selected criterion by one of the future engineers was typical for ecology but another future engineer, it was not because the first student originated from the region of operations of a mining company, where it was also talked about ecology in schools and media, as the strict rules apply to the operations of a global mining company (where competitive ecological extraction group was selected) specifically forced care about the environment while the local communities surrounding the extraction group paid close attention to the environment [39, 40, 41].

## 4 Conclusions

The initial diagnosis of the problem is not a new area of research. It is known at the lower level of education [42, 43]. This article highlights the need to extend the diagnosis to higher education. The case study presented concerned the university in Poland which is connected to the mining company, so the issue connected with the environment is highly recommended. The presented example of diagnosis concerns the ecology problem and the awareness of the students under the corporate social responsibility approach. Additionally, diagnoses in mathematical problems are presented to present the continued diagnosis from the lower education stage [44, 45, 46].

The main limitation of the proposed methodology is related to the time schedule for the occurrence for the occurrence of initial diagnoses. They should be performed at first classes, thus the whole procedure must be prepared before the semester starts. It demands to have all knowledge about the subject, preliminary knowledge (initial requirements) for a long time before the course starts. Also, the tools used must be verified regularly e.g. are the CSR standards still actual or some extensions are required [47].

In summary, the article indicated the possible area of initial diagnosis realization in universities. Implementation of initial diagnosis is some extent of innovation in higher education while ensuring a good practice for the high quality of education that is possible to be verified through its ongoing evaluation. The authors believe that this article will be the beginning of future research in universities.

## Declarations

### Author contribution statement

Elżbieta Jasińska: Conceived and designed the experiments; Performed the experiments; Analyzed and interpreted the data; Contributed reagents, materials, analysis tools or data; Wrote the paper.

Michał Jasińśki: Analyzed and interpreted the data; Wrote the paper.

### Funding statement

This work was supported by the Department of Operations Research and Business Intelligence [K43W08D12] and Department of Electrical Engineering Fundamentals [K38W05D02], Wroclaw University of Technology, Wroclaw, Poland.

### Data availability statement

Data included in article/supp. material/referenced in article.

### Declaration of interests statement

The authors declare no conflict of interest.

### Additional information

No additional information is available for this paper.
